# Supplementary material for: Content-rich biological network constructed by mining PubMed abstracts
Source: BMC Bioinformatics. 2004 Oct 8;5:147. doi: 10.1186/1471-2105-5-147 (PMC528731; doi:10.1186/1471-2105-5-147)
Supplement: Additional File 2 — The original results of the above study (non-essential files are deleted to keep the file size under the limit set by BMC bioinformatics). [file 1471-2105-5-147-S2.bz2 › chilibotAdditionalFile2/dip30/359ID9195914E5745/html/IL8_IL8RA.html]

 


 **IL8** and **IL8RA** 
  
Found 181 abstracts in PubMed, retrieved 30.  
 

 What does Google say? 
 PDF only 
| .edu only 

---

**Interactive relationship** (e.g. stimulation, inhibition, etc)

**Inhibitory relationship**- Analysis of inter relationships between CXCR1  [ **IL8RA** ]  and CXCR2 during internalization indicated that the exposure of cells that expressed both CXCR1  [ **IL8RA** ]  and CXCR2 to CXCL8  [ **IL8** ]  or CXCL6 resulted in decreased levels of CXCR1  [ **IL8RA** ]  internalization as compared to those in cells that expressed only CXCR1  [ **IL8RA** ] .  Ref: 12442335 Eur J Immunol, 2002
- In addition, a significant reduction of IL 8  [ **IL8** ]  CXCL8  [ **IL8** ]  mediated cell migration was reported in the presence of anti CXCR1  [ **IL8RA** ]  monoclonal antibody.  Ref: 12614207 Br J Haematol, 2003
- After phagocytosis of opsonized yeast, the expression of CXCR1  [ **IL8RA** ]  and CXCR2 was substantially down regulated and was accompanied by reduced Ca responses to corresponding ligands, IL 8  [ **IL8** ]  and neutrophil activating peptide 2 NAP  [ **IL8** ]  2.  Ref: 12239185 Blood, 2002
**Neutral relationship**- CXCR1  [ **IL8RA** ]  just binds IL 8  [ **IL8** ] .  Ref: 12365735 Fish Shellfish Immunol, 2002
- IL 8  [ **IL8** ]  Induced Migratory Responses through CXCR1  [ **IL8RA** ]  and CXCR2 Association with Phosphorylation and Cellular Redistribution of Focal Adhesion Kinase.  Ref: 12627953 Biochemistry, 2003
- IL 8  [ **IL8** ]  or CXCL8  [ **IL8** ]  activates the receptors CXCR1  [ **IL8RA** ]  IL 8RA and CXCR2 IL 8RB to induce chemotaxis in leukocytes, but only CXCR1  [ **IL8RA** ]  mediates cytotoxic and cross regulatory signals.  Ref: 12626541 J Immunol, 2003
- Interleukin 8  [ **IL8** ]  secreted by endothelial cells induces chemotaxis of melanoma cells through the chemokine receptor CXCR1  [ **IL8RA** ] .  Ref: 12738812 FASEB J< MedlineTA>FASEB J, 2003
- CXCL8  [ **IL8** ]  exerts potent angiogenic properties on endothelial cells through interaction with its cognate receptors CXCR1  [ **IL8RA** ]  and CXCR2.  Ref: 12496258 J Biol Chem, 2003
- The effects of IL 8  [ **IL8** ]  and its related ligands are mediated via two receptors, CXCR1  [ **IL8RA** ]  and CXCR2.  Ref: 12444152 J Immunol, 2002
- CXCR1  [ **IL8RA** ]  and CXCR2 mediate migratory activities in response to IL 8  [ **IL8** ]  and other ELR CXC chemokines e.g., GCP  [ **IL8** ]  2 and NAP  [ **IL8** ]  2.  Ref: 12627953 Biochemistry, 2003
- We recently engineered a series of high affinity CXCL8  [ **IL8** ]  IL 8  [ **IL8** ]  antagonists, one of which, CXCL8  [ **IL8** ]  3 73 K11R G31P, binds very strongly to neutrophils via the CXCR1  [ **IL8RA** ]  and CXCR2.  Ref: 12406656 Vet Immunol Immunopathol, 2002
- As CXCR1  [ **IL8RA** ]  and CXCR2 expression is differentially regulated in tissue specific endothelial cells and effects of IL 8  [ **IL8** ]  on intestinal endothelial cells are not defined, we characterized the potential IL 8  [ **IL8** ]  induced angiogenic mechanisms in primary cultures of human intestinal microvascular endothelial cells HIMEC and IL 8  [ **IL8** ]  receptor expression in human intestinal microvessels.  Ref: 12496258 J Biol Chem, 2003
- When stimulated by tumor necrosis factor alpha, the nonresponsive WM35 melanoma cells synthesize a higher level of CXCR1  [ **IL8RA** ]  and become chemotactic toward interleukin IL 8  [ **IL8** ] .  Ref: 12738812 FASEB J< MedlineTA>FASEB J, 2003

**Non-interactive relationship** (e.g. studied together, co-existance, homology, etc.)

- Thus in COPD, in addition to CXCL8  [ **IL8** ]  and CXCR1  [ **IL8RA** ] , CXCL5 and CXCR2 appear to play important roles in the airway neutrophilia characteristic of severe exacerbations.  Ref: 12857718 Am J Respir Crit Care MedAm J Respir Crit Care Med, 2003
- However, the contribution of polymorphisms of the IL 8  [ **IL8** ]  gene and its receptor genes CXCR 1  [ **IL8RA** ]  and CXCR  [ **IL8RA** ]  2 to human TB susceptibility remains untested.  Ref: 12870115 J Infect Dis, 2003
- In this study, we addressed the question of whether expression of the interleukin 8  [ **IL8** ]  receptors IL 8RA CXCR1  [ **IL8RA** ]  and IL 8RB CXCR2 is upregulated in H. pylori infected human gastric biopsy samples.  Ref: 12761119 Infect Immun, 2003
- To investigate the effects of chemokine receptor cross regulation in HIV 1 infection, monocytes and MAGIC5 and rat basophilic leukemia RBL 2H3 cell lines co expressing the interleukin 8  [ **IL8** ]  IL 8  [ **IL8** ]  or CXCL8  [ **IL8** ]  receptor CXCR1  [ **IL8RA** ]  and either CCR5 ACCR5 or CXCR4 ACXCR4 were generated.  Ref: 12594210 J Biol Chem, 2003
- Our data suggest that IL 8  [ **IL8** ]  directly enhanced endothelial cell proliferation, survival, and MMP expression in CXCR1  [ **IL8RA** ]  and CXCR2 expressing endothelial cells and regulated angiogenesis.  Ref: 12626597 J Immunol, 2003
- In the present study, we demonstrate that a potent and selective nonpeptide antagonist of human CXCR2 potently inhibits 125 I labeled human IL 8  [ **IL8** ]  binding to, and human IL 8  [ **IL8** ]  induced calcium mobilization mediated by, rabbit CXCR2 IC 50 = 40.5 and 7.7 nM, respectively, but not rabbit CXCR1  [ **IL8RA** ]  IC 50 = >1000 and 2200 nM, respectively.  Ref: 12444152 J Immunol, 2002
- In CXCR1  [ **IL8RA** ]  RBL cells this treatment resulted in FAK phosphorylation, in similar levels to those induced by activating concentrations of IL 8  [ **IL8** ] .  Ref: 12627953 Biochemistry, 2003
- Both IL 8  [ **IL8** ]  receptors, CXCR1  [ **IL8RA** ]  and CXCR2, are expressed extensively in the fetal intestine.  Ref: 12633567 Cytokine, 2002
- The interleukin 8  [ **IL8** ]  IL 8  [ **IL8** ]  CXCL8  [ **IL8** ]  receptor CXCR1  [ **IL8RA** ]  was expressed in 12 12 patients, the CXCR4 in 6 12 patients four CD3 ve and two CD3 ve and the CXCR3 in 3 12 patients one CD3 ve and two CD3 ve.  Ref: 12614207 Br J Haematol, 2003
- The immunohistochemical analysis using specific antibodies demonstrated that positive staining for IL 8  [ **IL8** ] , CXCR1  [ **IL8RA** ]  and CXCR2 in surgically resected human pancreatic cancer was 5 5 and 65%, respectively.  Ref: 12632066 Int J Oncol, 2003
- Cloning of the IL 8  [ **IL8** ]  receptor is important to study the function of IL 8  [ **IL8** ]  CXCR1  [ **IL8RA** ]  and CXCR2 in inflammation and signal transduction in fish.  Ref: 12365735 Fish Shellfish Immunol, 2002
- Antibodies against either IL 8  [ **IL8** ]  or CXCR1  [ **IL8RA** ]  inhibit melanoma transendothelial migration in a coculture assay by 30%.  Ref: 12738812 FASEB J< MedlineTA>FASEB J, 2002
- Thus far IL 8  [ **IL8** ]  and CXCR1  [ **IL8RA** ]  and CXCR2 proteins from rainbow trout have not been reported however, the sequence of a rainbow trout IL 8  [ **IL8** ]  has been noted GenBank Accession No.  Ref: 12365735 Fish Shellfish Immunol, 2002
- By comparison with the NSC and S COPD groups, neutrophilia and gene expression for epithelial derived neutrophil attractant 78 CXCL5, interleukin 8  [ **IL8** ]  CXCL8  [ **IL8** ] , CXCR1  [ **IL8RA** ]  and CXCR2 were each up regulated in the E COPD group p&lt.  Ref: 12857718 Am J Respir Crit Care MedAm J Respir Crit Care Med, 2002
- Biologic features, including IL 8  [ **IL8** ]  production, DNA binding activity, transactivation of NF kappaB, cell growth activity, cell viability, and the expression of IL 8  [ **IL8** ]  receptors CXCR1  [ **IL8RA** ]  and CXCR2 were analyzed.  Ref: 12216086 Cancer, 2002
- OBJECTIVE We sought to investigate the in vivo effect of bradykinin on the expression of the IL 8  [ **IL8** ]  receptors CXCR1  [ **IL8RA** ]  and CXCR2 in nasal cells.  Ref: 12532104 J Allergy Clin Immunol, 2003
- We examined the expression of IL 8  [ **IL8** ]  and its two receptors CXCR1  [ **IL8RA** ]  and CXCR2 in 40 surgically resected human pancreatic cancer tissues and in three different human pancreatic cancer cell lines PANC 1, MIAPaCa 2 and Capan 2.  Ref: 12632066 Int J Oncol, 2003
- Two IL 8  [ **IL8** ]  receptors, CXCR1  [ **IL8RA** ]  and CXCR2, have been identified in mammals 3 6.  Ref: 12365735 Fish Shellfish Immunol, 2002
- The stimulation of CXCR1  [ **IL8RA** ]  and CXCR2 by IL 8  [ **IL8** ]  concentrations that result in migratory activation induced focal adhesion kinase FAK phosphorylation in a G alpha i dependent manner.  Ref: 12627953 Biochemistry, 2003
- Because the chemotactic responses of neutrophils are impaired after phagocytosis, we explored the effect of phagocytic stimuli on the expression of interleukin 8  [ **IL8** ]  IL 8  [ **IL8** ]  receptors, CXCR1  [ **IL8RA** ]  and CXCR2, in human neutrophils.  Ref: 12239185 Blood, 2002
- The presence of wortmannin during receptor recycling inhibited CXCR1  [ **IL8RA** ]  and CXCR2 re expression following CXCL8  [ **IL8** ]  induced internalization, and resulted in a marked disruption of the proper organization of actin filaments.  Ref: 12442335 Eur J Immunol, 2002
- IL 8  [ **IL8** ]  activation of CXCR1  [ **IL8RA** ] , but not the IL 8  [ **IL8** ]  receptor CXCR2, cross phosphorylated CCR5 and CXCR4 and cross desensitized their responsiveness to RANTES regulated on activation normal T cell expressed and secreted CCL5 and stromal derived factor SDF 1 or CXCL12, respectively.  Ref: 12594210 J Biol Chem, 2003
- In the present study, we examined the expression of chemokine receptors CXCR1  [ **IL8RA** ]  and CXCR2 receptors for interleukin 8  [ **IL8** ]  and CCR5 receptor for RANTES regulated on activation, normal T cell expressed and secreted, macrophage inflammatory protein MIP 1alpha, and MIP 1beta in human endometrium.  Ref: 12606476 Biol Reprod, 2003
- There are at least two different IL 8  [ **IL8** ]  receptor types CXCR1  [ **IL8RA** ]  and CXCR2.  Ref: 12561382 Sheng Wu Yi Xue Gong Cheng Xue Za Zhi, 2002
- It is well known that chemokines and chemokine receptors, such as interleukin 8  [ **IL8** ]  and its receptors CXCR1  [ **IL8RA** ]  and CXCR2, play important roles in the pathogenesis of psoriasis.  Ref: 12823305 Clin Exp Dermatol, 2003
- Expression of the chemokine receptors CCR2, specific for MCP 1, and CXCR1  [ **IL8RA** ]  and CXCR2, specific for IL 8  [ **IL8** ] , were not altered by HCMV infection.  Ref: 12714640 Invest Ophthalmol Vis Sci, 2003
- The mutants generated showed that a for CXCL8  [ **IL8** ]  binding, the N terminus of CXCR1  [ **IL8RA** ]  and the second extra cellular loop of CXCR2 are determinant, the N terminus of CXCR2 is not sufficient and the transmembrane domain seven is probably involved.  Ref: 12628493 Biochem Pharmacol, 2003
- Transendothelial migration of neutrophils in response to CXCL7 or IL 8  [ **IL8** ]  CXCL8  [ **IL8** ]  was subject to modulation by CTAP III, but not CXCL4, as seen by drastic desensitization of the migratory response of neutrophils pre exposed to CTAP III, which was paralleled by selective down modulation of CXCR  [ **IL8RA** ]  2.  Ref: 12193731 J Immunol, 2002
- The investigation of expression in IL 8  [ **IL8** ]  receptors, CXCR1  [ **IL8RA** ]  and CXCR2, revealed that the expression of both receptors was enhanced remarkably by curcumin.  Ref: 12216086 Cancer, 2002
